# Supplementary material for: Evolutionary measures show that recurrence of DCIS is distinct from progression to breast cancer
Source: Breast Cancer Res. 2025 Mar 21;27:43. doi: 10.1186/s13058-025-01966-2 (PMC11929273; doi:10.1186/s13058-025-01966-2)
Supplement: Supplementary file 1 — Additional file1 (PDF 877 KB) [file 13058_2025_1966_MOESM1_ESM.pdf]

Supplementary Materials for the Manuscript:

Evolutionary Measures Show that Recurrence of DCIS is Distinct from Progression to Breast Cancer

Angelo Fortunato[1,2*], Diego Mallo[1,2*], Luis Cisneros[1*], Lorraine M. King[3], Aziz Khan[4,5], Christina Curtis[4,5,6], Marc D. Ryser[3], Joseph Y. Lo[3], Allison Hall[3], Jeffrey R. Marks[3],
E. Shelley Hwang[3**] and Carlo C. Maley[1,2**]

Table of contents

Supplementary Text 1: Immunohistochemistry Distances **3**
IHC measurements 3
Mean of Intensity Score (MIS) 3
Earth Mover's Distance (EMD) 3
Cumulative Density Index (CDI) 4
Controlling for triple-negative breast cancer 4
References 5
Supplementary Figures 6
Figure S1. Cross-sectional SNV divergence within synchronous DCIS and between synchronous DCIS and IDC. 6
Figure S2. Cross-sectional phenotypic characterization. 7
Figure S3. Cross-sectional phenotypic between-sample divergence. 8
Figure S4. Cross-sectional phenotypic within-sample divergence. 9
Figure S5. ER longitudinal phenotypic characterization in all patients. 10
Figure S6. Longitudinal phenotypic divergence. 11
Figure S7. Event-free survival curves of patients stratified by SNV burden: alternative clinical outcomes. 12
Figure S8. Associations with time to clinical outcome: alternative clinical outcomes. 13
Figure S9. Associations with time to clinical outcome: alternative clinical margin covariate. 14
Figure S10. Associations with time to clinical outcome without covariate imputation. 15
Figure S11. Associations with time to clinical outcome without covariate imputation: alternative clinical outcomes. 16
Figure S12. Associations with time to clinical outcome without covariate imputation: alternative clinical margin covariate. 17
Supplementary Tables **18**
Table S1. Breakdown of the number of patients for each monoclonal immunohistochemical marker in the cross-sectional study. 18

Table S2. Breakdown of the number of patients for each monoclonal immunohistochemical marker in the longitudinal study. 19
Table S3. Breakdown of the number of patients for each monoclonal immunohistochemical marker in the longitudinal study restricted to ER+ patients. 19
Table S4. Clinical variables considered in the proportional hazard regressions of the longitudinal study. 20
Table S5. DAVID functional annotation clustering results. 21
Table S6. PANTHER overrepresentation test results using the Fisher test and FDR correction. 24
Table S7. Univariate proportional hazard regressions of Time to Recurrence. 25
Table S8. Univariate proportional hazard regressions of time to progression from nonprogressors 25
Table S9. Univariate proportional hazard regressions of time to non-invasive recurrence 25
Table S10. Univariate proportional hazard regressions of time to progression 26

Supplementary Text 1: Immunohistochemistry Distances

IHC measurements

An IHC profile is a set of categories indicating the percentage of the slide presenting basic levels of staining intensity: {C_0, C_1, C_2, C_3}, where C_0 is the fraction of the block with no staining, and C_1, C_2, and C_3 are the fractions of the sample with low, medium and high staining intensity.

Mean of Intensity Score (MIS)

For an IHC marker profile, the total intensity score is defined as the weighted sum of intensity values normalized by the maximum possible staining (meaning the IHC profile where the whole slide has high staining intensity):

$$I = \left(\frac{1}{100n}\right)\sum_{i=0}^{n} i \cdot C_i$$

Where C_i is the fraction of the block with staining level *i*. The Mean of Intensity Score is the mean across blocks for each patient, measuring the typical observed staining intensity for that marker.

Earth Mover's Distance (EMD)

We consider the differences in the IHC profile between blocks for each patient to assess (distant) phenotypic heterogeneity in the tissue. EMD is a Wasserstein metric representing the minimum cost of turning one profile into another (1). The general cost function is computed as the product of the fraction of the profile and the distance that fraction is moved (measured as the number of categorical steps required to turn one profile into the other). For instance:

1. The score between profiles {100, 0, 0 , 0} and {0,100,0,0} is
   EMD({100, 0, 0 , 0}, {0,100,0,0}) = 1.0 x 1 = 1,
   because all the units are moved one level up.

2. In the same way,
   EMD({100, 0, 0 , 0}, {50,0,50,0}) = 0.5 x 2 = 1
   because half the units are moved two levels.

3. And
   EMD({100, 0, 0 , 0}, {0,0,0,100}) = 1.0x3 = 3,
   which is the maximum possible distance for this metric.

EMD is a pairwise metric that returns a distance measure, defined between zero, for identical profiles and (n-1) for maximally dissimilar profiles with n levels. We used the package *emdist* (version 0.3-2) implemented in R to evaluate this metric.

Cumulative Density Index (CDI)

We consider a measure of within-sample heterogeneity defined as follows:

$$\text{CDI} = 1 - \left|\frac{\sum_{i=0}^{n}(S_i - L_i)}{\sum_{i=0}^{n}(1 - L_i)}\right|$$

with $S_i = \sum_{i=0}^{i} P_j$ the cumulative values of the normalized profile, $L_i = \frac{i+1}{n+1}$ the cumulative values of a uniform profile (i.e., {0.25, 0.25, 0,25, 0.25} giving a cumulative {0.25, 0.50, 0.75, 1.0}). The denominator term is the same as the area under the cumulative uniform profile, and the numerator term is the area between the observed cumulative profile and the uniform one. With this definition, CDI represents how close to a uniform distribution the observed profile is. If the observed profile is uniform, then CDI=1, and if the observed profile is one of the extremes (e.g., {100, 0, 0, 0} or {0,0,0,100}), then CDI=0 (see Figure ST1).

This score is particularly sensitive to distinguishing extreme staining cases (i.e., all-or-nothing cases) versus cases with more diverse profiles. Values of CDI close to 0 indicate **less heterogeneity**, while values close to 1 signify more diverse profiles. A uniform profile, CDI=1, would indicate an equal representation of staining intensity in all levels, which is the maximum level of heterogeneity possible in the sample.

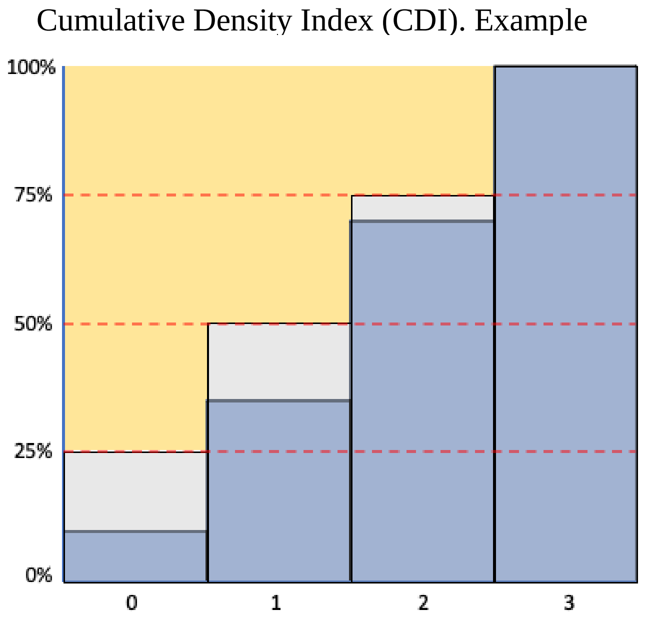

Fig ST1: Grey boxes represent the difference between the observed cumulative profile (blue) and a reference uniform cumulative profile ({0.25, 0.50, 0.75, 1.00}). The yellow area represents the area under the uniform profile. The value of CDI is given by the ratio between the grey area and the yellow area.

Controlling for triple-negative breast cancer

To control for the possible confounding effect of triple-negative breast cancer samples, which are known to have an elevated risk of subsequent breast events, we did an additional analysis in which we eliminated cases with negative ER status (PR and HER2 status were not known for many cases) for all of the regular IHC staining, additional standard clinical IHC, and RNA. Their phenotypic characterization and divergence were not qualitatively different, and their clinical outcome prediction results were very similar.

References

1. Orlova DY, Zimmerman N, Meehan S, Meehan C, Waters J, Ghosn EEB, et al. Earth Mover's Distance (EMD): A True Metric for Comparing Biomarker Expression Levels in Cell Populations. PLoS One [Internet]. 2016;11:e0151859. Available from: http://dx.doi.org/10.1371/journal.pone.0151859

Supplementary Figures

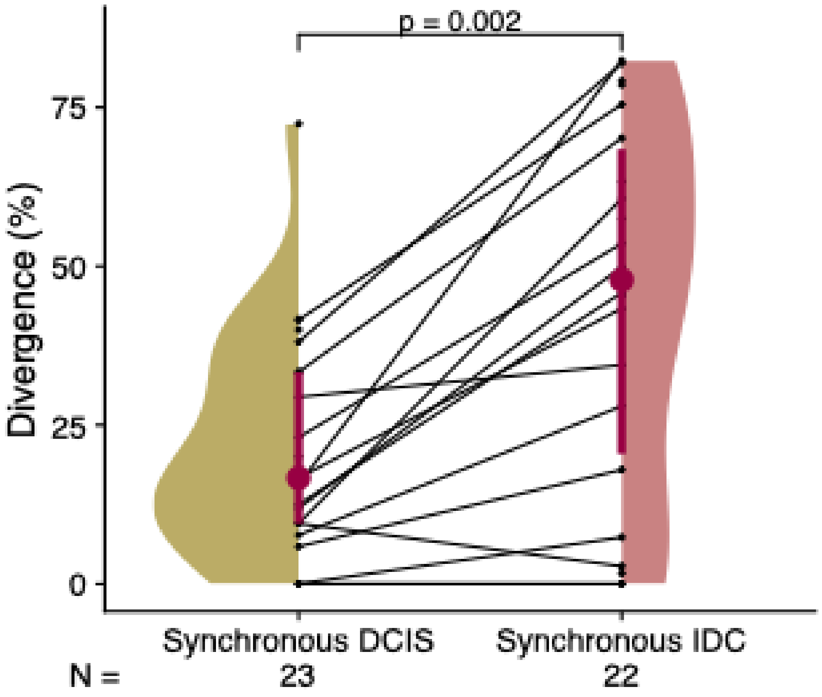

Figure S1. Cross-sectional SNV divergence within synchronous DCIS and between synchronous DCIS and IDC.
Distribution of SNV genetic divergence (percentage of private mutations) per synchronous patient, calculated within DCIS samples (Synchronous DCIS) or between DCIS and IDC samples (Synchronous IDC). Synchronous IDC data points are the mean of two comparisons between 2 DCIS samples and 1 IDC sample. Paired-samples sign test. Interquartile range (vertical line) and median (point) in burgundy. N: number of data points.

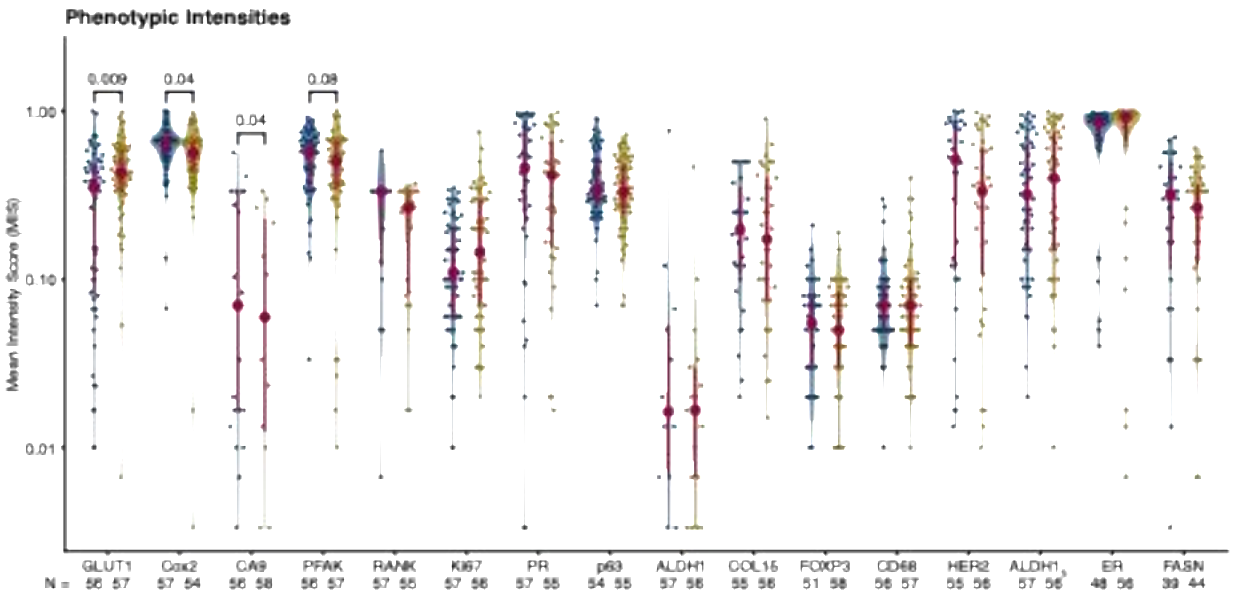

Figure S2. Cross-sectional phenotypic characterization.
Distribution of intensity scores of one sample per patient sampled at random for each patient and IHC marker (unadjusted p-values). Unadjusted pairwise Mann-Whitney U p-values shown if p ≤ 0.1. Interquartile range (vertical line) and median (point) in burgundy. N: number of patients.

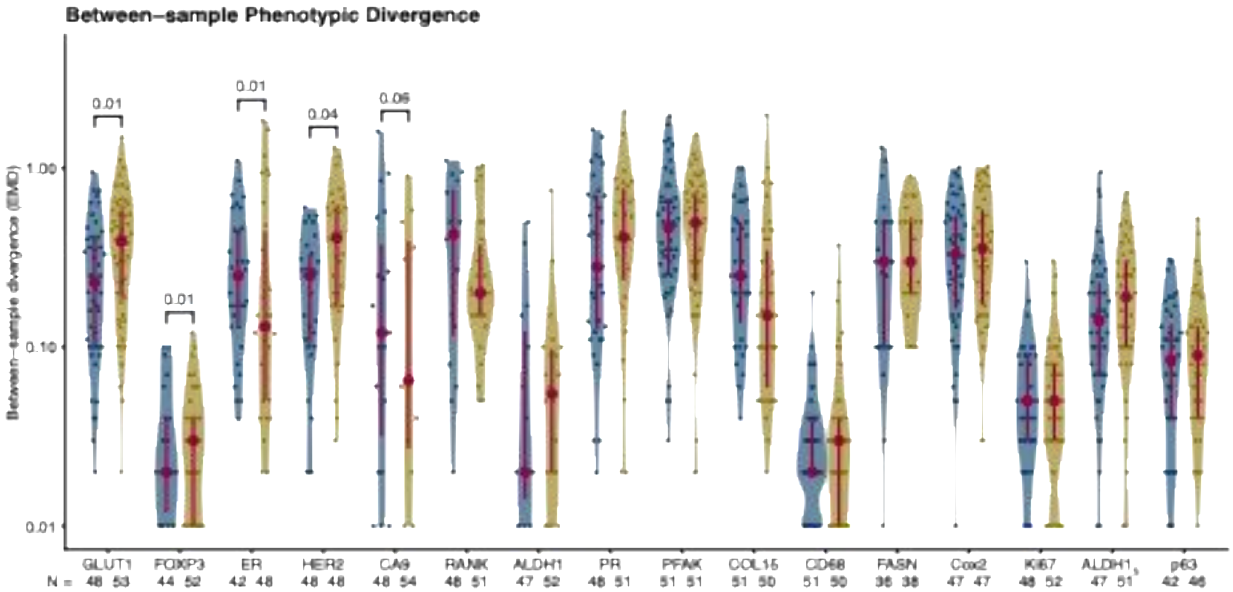

Figure S3. Cross-sectional phenotypic between-sample divergence.
Distribution of Earth Mover's Distances (EMDs) for each patient and IHC marker. Unadjusted pairwise Mann-Whitney U p-values shown if $p \leq 0.1$. Interquartile range (vertical line) and median (point) in burgundy. N: number of patients.

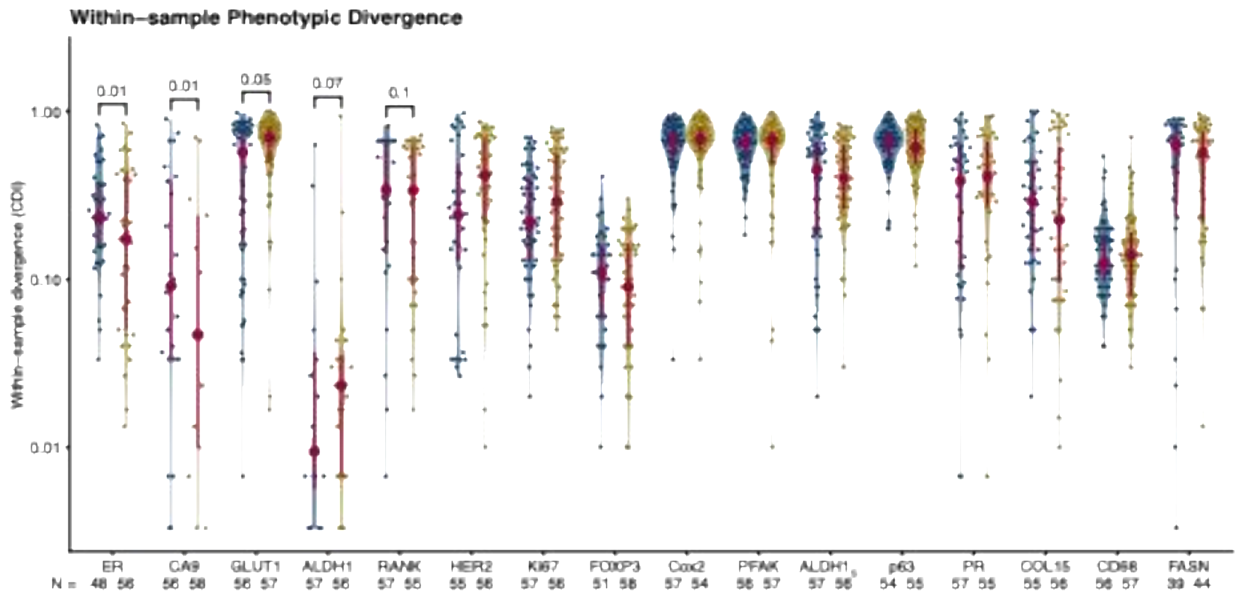

Figure S4. Cross-sectional phenotypic within-sample divergence.
Distribution of Cumulative Density Indices (CDIs) for each patient and IHC marker. Unadjusted pairwise Mann-Whitney U p-values shown if $p \leq 0.1$. Interquartile range (vertical line) and median (point) in burgundy. N: number of patients.

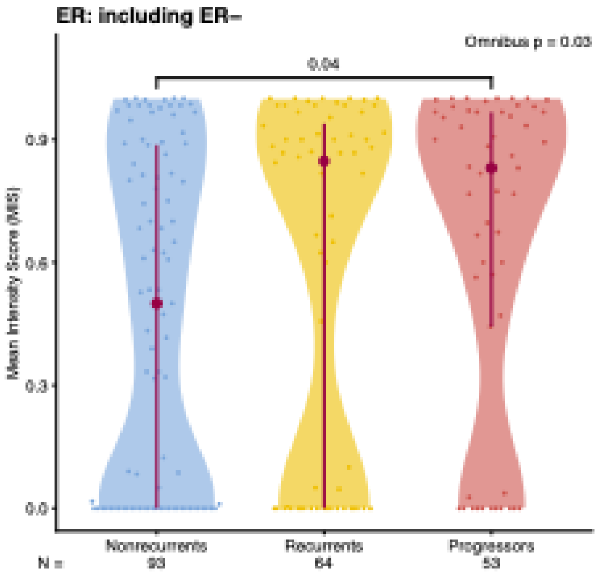

Figure S5. ER longitudinal phenotypic characterization in all patients.
In contrast with Fig 5B, ER- patients are included here. Distribution of mean normalized intensities (MIS) per patient (see Methods). Omnibus test: Kruskal-Wallis Rank Sum, Post-hoc test: Dunn's test with control for multiple tests using the Holm-Šidák adjustment. Statistically significant differences between groups shown if adjusted p ≤ 0.05. Interquartile range (vertical line) and median (point) in burgundy. N: number of patients.

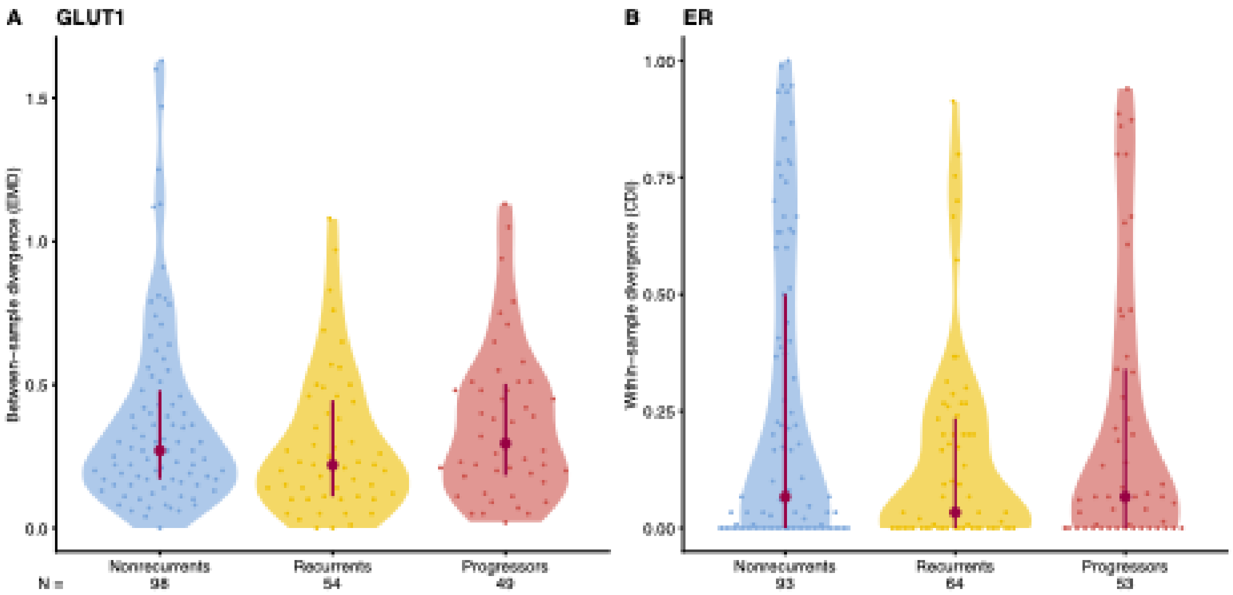

Figure S6. Longitudinal phenotypic divergence.

Distribution of measures of divergence per patient and marker. A, GLUT1 between-sample divergence, Earth Mover's Distance (EMD). B, ER within-sample divergence, Cumulative Density Index (CDI). Omnibus test: Kruskal-Wallis Rank Sum, Post-hoc test: Dunn's test with control for multiple tests using the Holm-Šidák adjustment. Statistically significant differences between groups shown if adjusted $p \leq 0.05$. Interquartile range (vertical line) and median (point) in burgundy. N: number of patients.

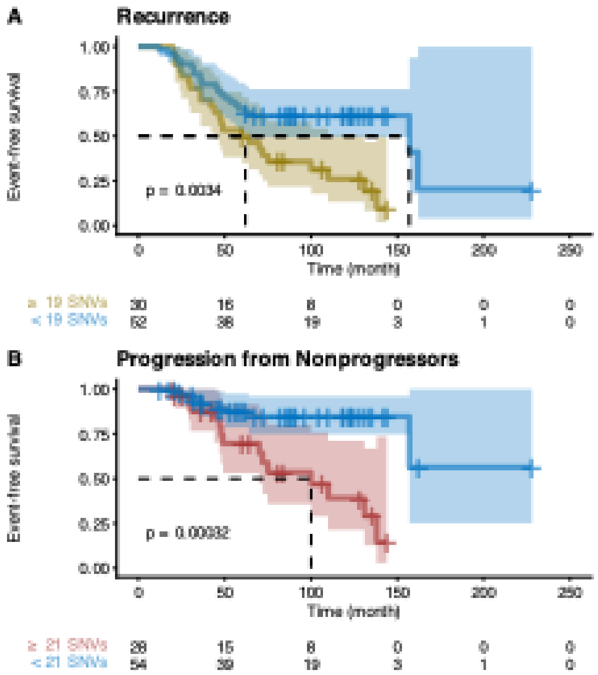

Figure S7. Event-free survival curves of patients stratified by SNV burden: alternative clinical outcomes.

Alternative to Fig. 6 using different clinical outcomes: recurrence instead of non-invasive recurrence and progression (right-censoring *recurrents* at time of recurrence) instead of progression (removing *recurrents*). Kaplan-Meier plots of stratified patients. **A:** Recurrence-free survival. **B:** Progression-free survival (right-censoring *recurrents* at recurrence time). SNV burden thresholds maximize Youden's J statistic of the outcomes. Log-rank test. The table below the Kaplan-Meier plot shows the number of samples at risk at different times.

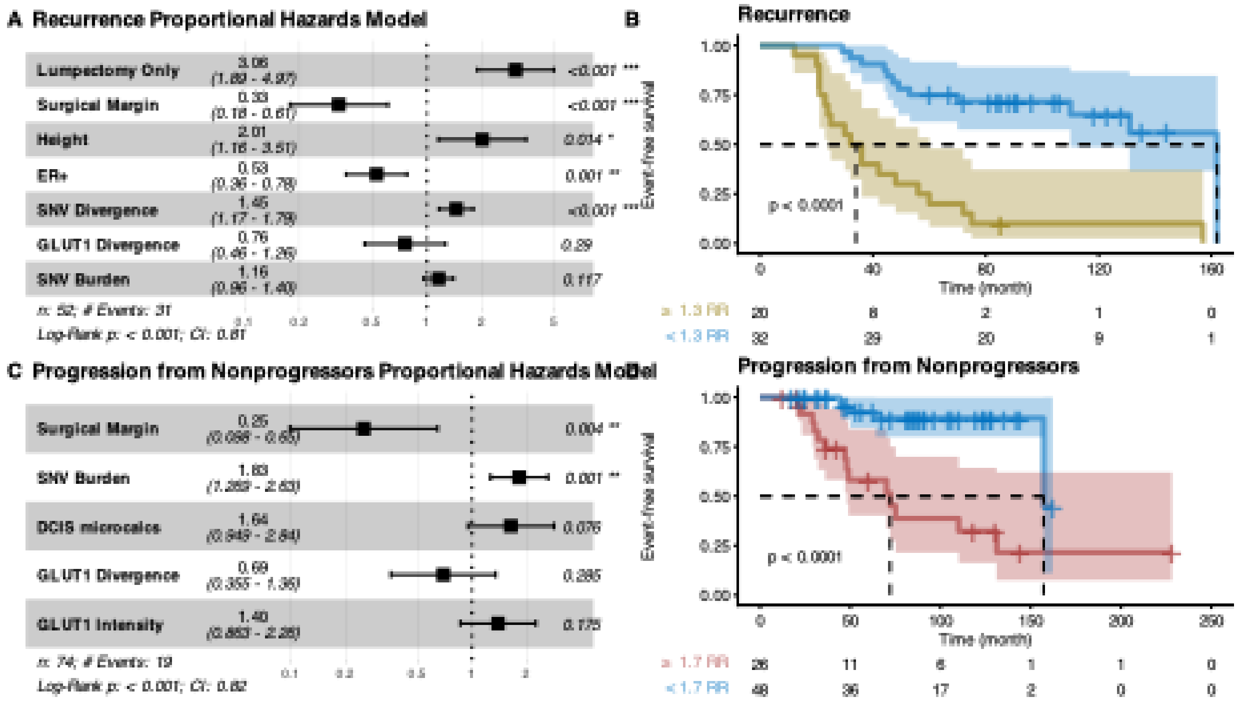

Figure S8. Associations with time to clinical outcome: alternative clinical outcomes. Alternative to Fig. 7 using different clinical outcomes: recurrence instead of non-invasive recurrence and progression (right-censoring *recurrents* at time of recurrence) instead of progression (removing *recurrents*). Forest plots describing proportional hazard regressions using variables selected with LASSO (**A**, **C**) and corresponding Kaplan-Meier plots of patients stratified by the relative risk threshold that maximizes Youden's J statistic of the outcomes (**B**, **D**). **A-B**: Recurrence-free survival. **C-D**: Progression-free survival (right-censoring *recurrents* at recurrence time). Log-rank test. Tables below Kaplan-Meier plots show the number of samples at risk at different times.

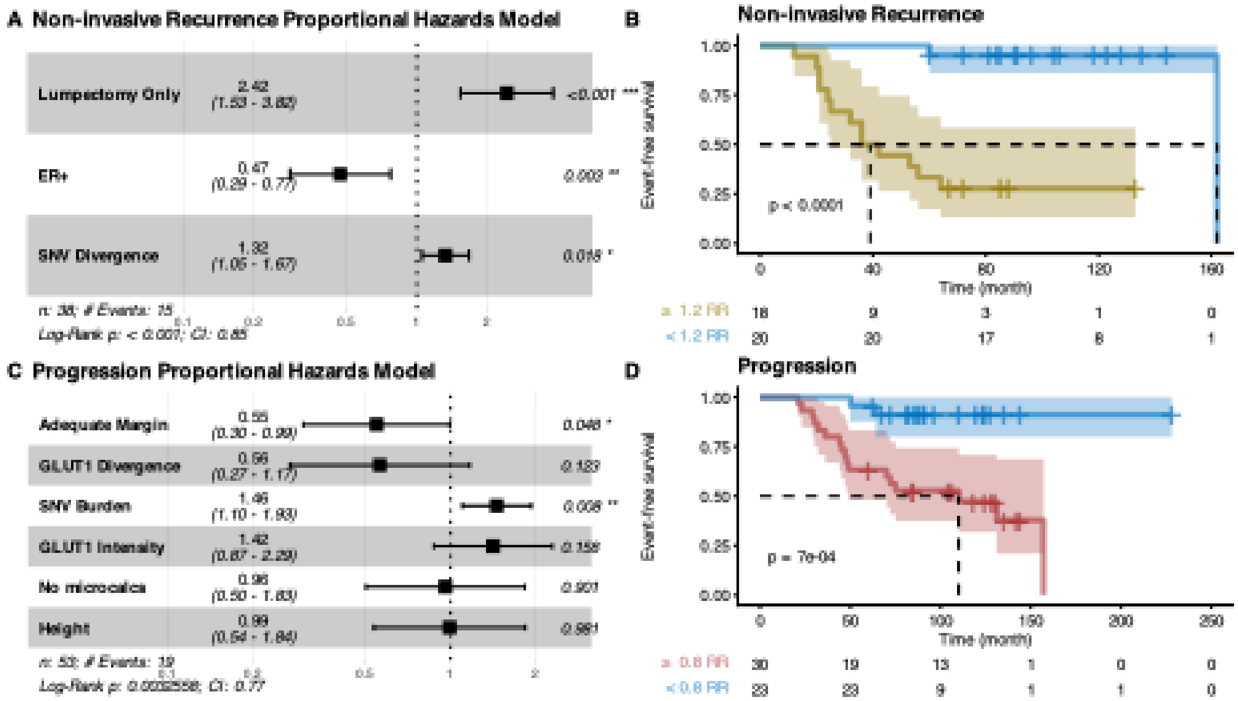

Figure S9. Associations with time to clinical outcome: alternative clinical margin covariate.

Alternative to Fig. 7 using a 2mm threshold to encode the surgical margin covariate. Forest plots describing proportional hazard regressions using variables selected with LASSO (**A**, **C**) and corresponding Kaplan-Meier plots of patients stratified by the relative risk threshold that maximizes Youden's J statistic of the outcomes (**B**, **D**). **A-B**: Non-invasive-recurrence-free survival. **C-D**: Progression-free survival. Log-rank test. Tables below Kaplan-Meier plots show the number of samples at risk at different times.

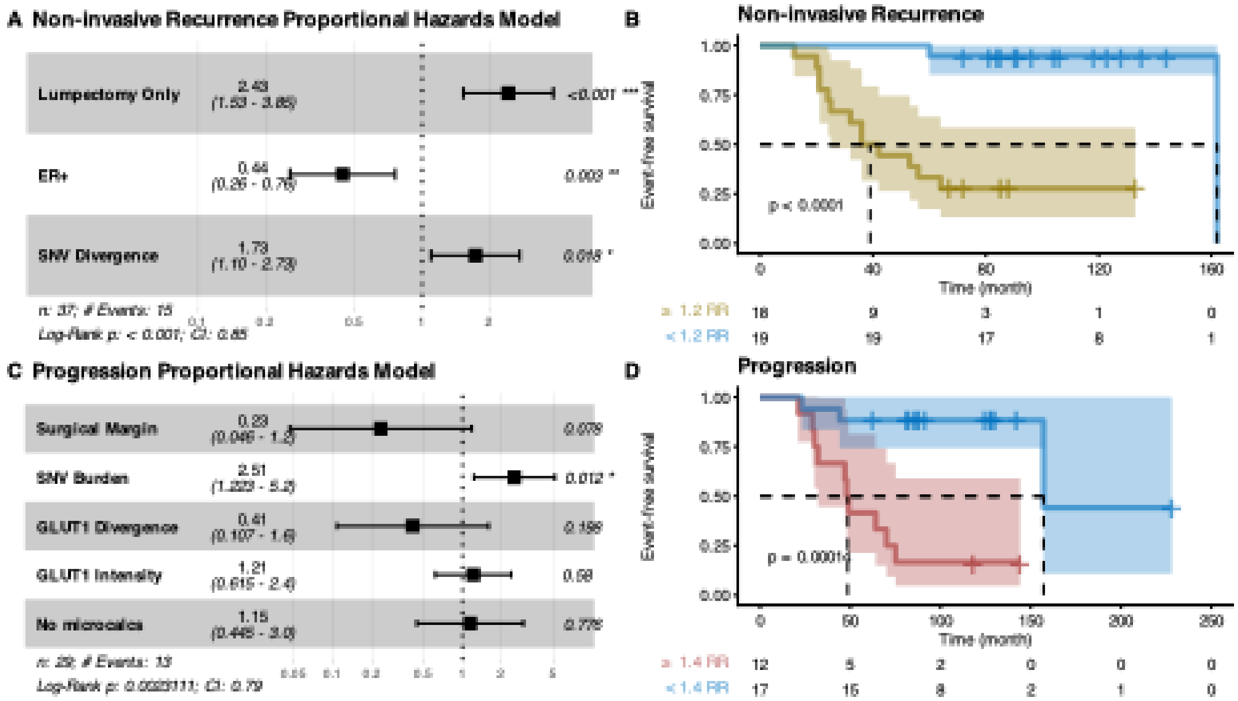

Figure S10. Associations with time to clinical outcome without covariate imputation. Forest plots describing proportional hazard regressions using variables selected with LASSO (A, C) and corresponding Kaplan-Meier plots of patients stratified by the relative risk threshold that maximizes Youden's J statistic of the outcomes (B, D). A-B: Recurrence-free survival. C-D: Progression-free survival. Log-rank test. Tables below Kaplan-Meier plots show the number of samples at risk at different times.

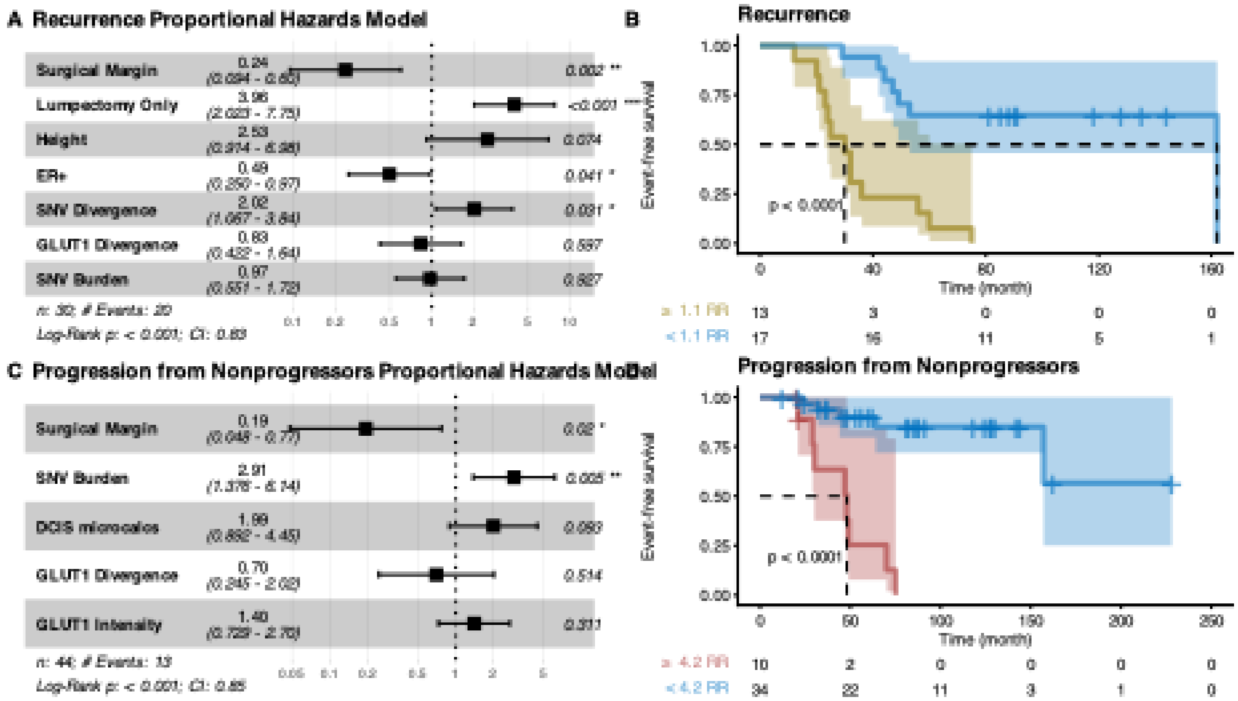

Figure S11. Associations with time to clinical outcome without covariate imputation: alternative clinical outcomes.

Alternative to Fig. S7 using different clinical outcomes: recurrence instead of non-invasive recurrence and progression (right-censoring *recurrents* at time of recurrence) instead of progression (removing *recurrents*). Forest plots describing proportional hazard regressions using variables selected with LASSO (A, C) and corresponding Kaplan-Meier plots of patients stratified by the relative risk threshold that maximizes Youden's J statistic of the outcomes (B, D). A-B: Recurrence-free survival. C-D: Progression-free survival (right-censoring *recurrents* at recurrence time). Log-rank test. Tables below Kaplan-Meier plots show the number of samples at risk at different times.

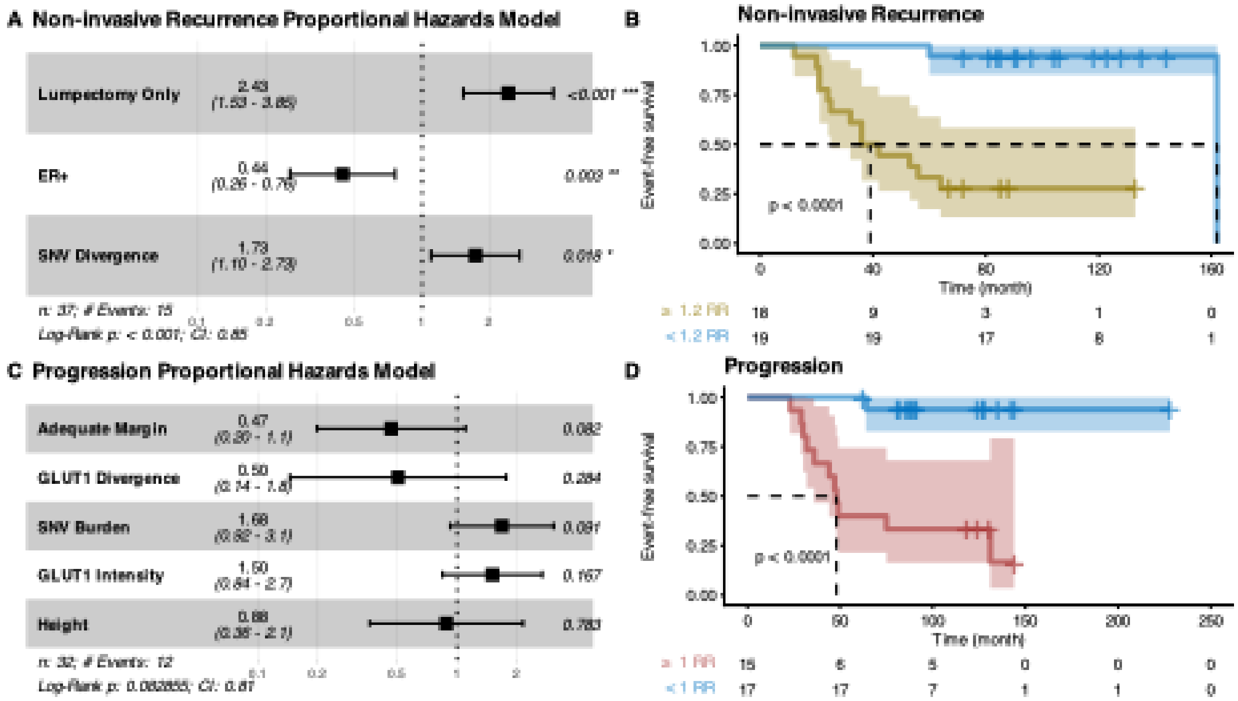

Figure S12. Associations with time to clinical outcome without covariate imputation: alternative clinical margin covariate.

Alternative to Fig. S7 using a 2mm threshold to encode the surgical margin covariate. Forest plots describing proportional hazard regressions using variables selected with LASSO (**A**, **C**) and corresponding Kaplan-Meier plots of patients stratified by the relative risk threshold that maximizes Youden's J statistic of the outcomes (**B**, **D**). **A-B**: Non-invasive-recurrence-free survival. **C-D**: Progression-free survival. Log-rank test. Tables below Kaplan-Meier plots show the number of samples at risk at different times.

Supplementary Tables

Table S1. Breakdown of the number of patients for each monoclonal immunohistochemical marker in the cross-sectional study.

Categories	IHC	Pure	Synchronous
Intensity (class 0-3: NULL, S, M, L)	ALDH1	47	52
	CA9	48	54
	ER	42	48
	FASN	36	38
	GLUT1	48	53
	COX2	47	47
	HER2	48	48
	PFAK	51	51
	PR	48	51
	RANK	48	51
	COL15	51	50
Signal (class 0-1: neg, pos)	ALDH1 Stroma	47	51
	CD68 macrophage index	51	50
	Ki67	48	52
	FOXP3	44	52
	P63	42	46

Table S2. Breakdown of the number of patients for each monoclonal immunohistochemical marker in the longitudinal study.

Marker	Cohort	Counts
ER	Nonrecurrents	92
ER	Recurrents	65
ER	Progressors	53
GLUT1	Nonrecurrents	97
GLUT1	Recurrents	55
GLUT1	Progressors	49

Table S3. Breakdown of the number of patients for each monoclonal immunohistochemical marker in the longitudinal study restricted to ER+ patients.

Marker	Cohort	Counts
ER	Nonrecurrents	70
ER	Recurrents	50
ER	Progressors	43
GLUT1	Nonrecurrents	75
GLUT1	Recurrents	43
GLUT1	Progressors	39

Table S4. Clinical variables considered in the proportional hazard regressions of the longitudinal study.

Name	Type	Unit/Categories	Notes
**Age at DCIS Diagnosis**	Integer	year	
**Menopausal Status**	Categorical	Pre, Post	
**Race**	Categorical	White, Black, Other	
**Height**	Float	cm	
**Weight**	Float	kg	
**BMI**	Float	kg/m^2	
**Axillary Dissection (at surgical treatment)**	Categorical	None, Sentinel Lymph Node Biopsy, Axillary Dissection	
**Number of Nodes Examined (at surgical treatment)**	Integer	number of nodes	Node fragment recoded to 1
**DCIS at Surgical Margin**	Logical		
**Surgical Margin**	Float	mm	Most < 10mm were measured precisely. The rest were completed from a categorical variable (<1, 1-10, >10) with 0.5, 2.5, and 10 values
**DCIS size**	Float	mm	
**Nuclear Grade**	Categorical	1, 2, 3	
**Necrosis Type**	Categorical	Comedo, non-comedo, Other, None	
**Microcalcification Type**	Categorical	DCIS, Benign, Both	
**ER**	Logical		
**PR**	Logical		
**DCIS Treatment**	Categorical	Lumpectomy only, Lumpectomy w Radiation, Mastectomy	
**Hormonal Therapy**	Logical		

Table S5. DAVID functional annotation clustering results.

Knowledgebase v2024q1. # = count, Fold = Enrichment fold.

Category	Term	#	%	PValue	Genes	List Total	Pop Hits	Pop Total	Fold	FDR
**Nonrecurrents**										
**None**										
**Recurrents**										
**Annotation Cluster 1**	**Enrichment Score: 3.5**									
**Category**	**Term**	**#**	**%**	**PValue**	**Genes**	**List Total**	**Pop Hits**	**Pop Total**	**Fold**	**FDR**
GOTERM_MF_DIRECT	GO:0033038~bitter taste receptor activity	4	5,3	8,2E-05	TAS2R30, TAS2R31, TAS2R43, TAS2R46 (*Gene set 1*)	71	23	18883	46,3	0,015
INTERPRO	IPR007960:TAS2R	4	5,3	1,3E-04	*Gene set 1*	74	28	20603	39,8	0,037
GOTERM_BP_DIRECT	GO:0050909~sensory perception of taste	4	5,3	1,7E-04	*Gene set 1*	64	33	19256	36,5	0,094
“”	GO:0001580~detection of chemical stimulus involved in sensory perception of bitter taste	4	5,3	3,0E-04	*Gene set 1*	64	40	19256	30,1	0,094
UP_KW_BIOLOGICAL_PROCESS	KW-0919~Taste	4	5,3	3,3E-04	*Gene set 1*	42	38	11447	28,7	0,007
KEGG_PATHWAY	hsa04742:Taste transduction	4	5,3	0,003	*Gene set 1*	31	86	8662	13,0	0,439
**Progressors**										
**Annotation Cluster 1**	**Enrichment Score: 3.6**									
**Category**	**Term**	**#**	**%**	**PValue**	**Genes**	**List Total**	**Pop Hits**	**Pop Total**	**Fold**	**FDR**
UP_SEQ_FEATURE	REPEAT:Spectrin 18	4	2,4	2,91E-05	SPTA1, SPTBN5, DST, SPTAN1 (*Gene set 2*)	169	8	20562	60,83	0,0110
“”	REPEAT:Spectrin 19	4	2,4	2,91E-05	*Gene set 2*	169	8	20562	60,83	0,0110
“”	REPEAT:Spectrin 20	4	2,4	2,91E-05	*Gene set 2*	169	8	20562	60,83	0,0110
“”	REPEAT:Spectrin 15	4	2,4	1,12E-04	*Gene set 2*	169	12	20562	40,56	0,0136
“”	REPEAT:Spectrin 16	4	2,4	1,12E-04	*Gene set 2*	169	12	20562	40,56	0,0136
“”	REPEAT:Spectrin 17	4	2,4	1,12E-04	*Gene set 2*	169	12	20562	40,56	0,0136
“”	REPEAT:Spectrin 10	4	2,4	1,44E-04	*Gene set 2*	169	13	20562	37,44	0,0136
“”	REPEAT:Spectrin 11	4	2,4	1,44E-04	*Gene set 2*	169	13	20562	37,44	0,0136

“”	REPEAT:Spectrin 12	4	2,4	1,44E-04	*Gene set 2*	169	13	20562	37,44	0,0136
“”	REPEAT:Spectrin 13	4	2,4	1,44E-04	*Gene set 2*	169	13	20562	37,44	0,0136
“”	REPEAT:Spectrin 14	4	2,4	1,44E-04	*Gene set 2*	169	13	20562	37,44	0,0136
“”	REPEAT:Spectrin 7	4	2,4	1,83E-04	*Gene set 2*	169	14	20562	34,76	0,0137
“”	REPEAT:Spectrin 8	4	2,4	1,83E-04	*Gene set 2*	169	14	20562	34,76	0,0137
“”	REPEAT:Spectrin 9	4	2,4	1,83E-04	*Gene set 2*	169	14	20562	34,76	0,0137
“”	REPEAT:Spectrin 5	4	2,4	2,27E-04	*Gene set 2*	169	15	20562	32,44	0,0151
“”	REPEAT:Spectrin 6	4	2,4	2,27E-04	*Gene set 2*	169	15	20562	32,44	0,0151
“”	REPEAT:Spectrin 3	4	2,4	7,36E-04	*Gene set 2*	169	22	20562	22,12	0,0416
“”	REPEAT:Spectrin 4	4	2,4	7,36E-04	*Gene set 2*	169	22	20562	22,12	0,0416
INTERPRO	IPR002017:Spectrin_repeat	4	2,4	9,18E-04	*Gene set 2*	167	24	20603	20,56	0,1980
UP_SEQ_FEATURE	REPEAT:Spectrin 1	4	2,4	0,001	*Gene set 2*	169	26	20562	18,72	0,0596
“”	REPEAT:Spectrin 2	4	2,4	0,001	*Gene set 2*	169	26	20562	18,72	0,0596
INTERPRO	IPR018159:Spectrin/alpha-actinin	4	2,4	0,002	*Gene set 2*	167	30	20603	16,45	0,2572
SMART	SM00150:SPEC	4	2,4	0,003	*Gene set 2*	110	29	10690	13,40	0,3468
**Annotation Cluster 2**	**Enrichment Score: 3.3**									
**Category**	**Term**	**#**	**%**	**PValue**	**Genes**	**List Total**	**Pop Hits**	**Pop Total**	**Fold**	**FDR**
KEGG_PATHWAY	hsa05213:Endometrial cancer	6	3,6	8,53E-05	PIK3CA, ERBB2, PTEN, AKT1, PIK3R2, TP53 (*Gene set 3*)	69	58	8662	13,0	0,006
KEGG_PATHWAY	hsa05230:Central carbon metabolism in cancer	6	3,6	2,10E-04	*Gene set 3*	69	70	8662	10,8	0,010
KEGG_PATHWAY	hsa05215:Prostate cancer	6	3,6	9,48E-04	*Gene set 3*	69	97	8662	7,8	0,021
KEGG_PATHWAY	hsa05224:Breast cancer	6	3,6	0,006	*Gene set 3*	69	147	8662	5,1	0,054
**Annotation Cluster 3**	**Enrichment Score: 2.2**									
**Category**	**Term**	**#**	**%**	**PValue**	**Genes**	**List Total**	**Pop Hits**	**Pop Total**	**Fold**	**FDR**
KEGG_PATHWAY	hsa05223:Non-small cell lung cancer	5	3,0	0,002	PIK3CA, ERBB2, AKT1, PIK3R2,	69	72	8662	8,7	0,036

					TP53 (*Gene set 4*)					
KEGG_PATHWAY	hsa05212:Pancreatic cancer	5	3,0	0,003	*Gene set 4*	69	76	8662	8,3	0,038
KEGG_PATHWAY	hsa05226:Gastric cancer	5	3,0	0,029	*Gene set 4*	69	149	8662	4,2	0,147
**Annotation Cluster 4**	**Enrichment Score: 2.1**									
**Category**	**Term**		**%**	**PValue**	**Genes**	**List Total**	**Pop Hits**	**Pop Total**	**Fold**	**FDR**
KEGG_PATHWAY	hsa05218:Melanoma	5	3,0	0,002	PIK3CA, PTEN, AKT1, PIK3R2, TP53 (*Gene set 5*)	69	72	8662	8,7	0,036
KEGG_PATHWAY	hsa05214:Glioma	5	3,0	0,003	*Gene set 5*	69	75	8662	8,4	0,038
KEGG_PATHWAY	hsa04071:Sphingolipid signaling pathway	5	3,0	0,015	*Gene set 5*	69	121	8662	5,2	0,094
KEGG_PATHWAY	hsa05225:Hepatocellular carcinoma	5	3,0	0,043	*Gene set 5*	69	168	8662	3,7	0,178

Table S6. PANTHER overrepresentation test results using the Fisher test and FDR correction.

**Nonrecurrents**						
PANTHER Pathways	None					
**Recurrents**						
PANTHER Pathways	None					
**Progressors**						
**PANTHER Pathways**	**H. sapiens REFLIST (20592)**	**Input (175)**	**Expected**	**Enrichment (fold)**	**p-val**	**FDR**
**Hypoxia response via HIF activation (P00030)**	30	5	.25	20.01	5.02E-06	8.09E-04
**Hedgehog signaling pathway (P00025)**	20	3	.17	18.05	6.18E-04	1.66E-02
**Insulin/IGF pathway-protein kinase B signaling cascade (P00033)**	38	5	.32	15.48	1.67E-05	8.99E-04
**p53 pathway feedback loops 2 (P04398)**	52	6	.44	13.58	5.09E-06	4.10E-04
**Axon guidance mediated by netrin (P00009)**	35	3	.30	10.09	3.23E-03	5.21E-02
**Endothelin signaling pathway (P00019)**	84	6	.71	8.40	8.12E-05	3.27E-03
**PI3 kinase pathway (P00048)**	56	4	.48	8.40	1.31E-03	2.64E-02
**p53 pathway (P00059)**	88	6	.75	8.02	1.05E-04	3.39E-03
**VEGF signaling pathway (P00056)**	69	4	.59	7.22	2.84E-03	5.07E-02
**Ras Pathway (P04393)**	73	4	.62	6.45	3.48E-03	5.09E-02
**EGF receptor signaling pathway (P00018)**	138	6	1.17	5.12	1.18E-03	2.71E-02
**Apoptosis signaling pathway (P00006)**	119	5	1.01	5.34	3.51E-03	4.70E-02

Table S7. Univariate proportional hazard regressions of Time to Recurrence.

Variable	p.val	adj.p.val	C	HR (95% CI)
SNV burden	0.0022	0.017	0.58	1.5 (1.2 - 2)
CNA divergence	0.023	0.16	0.57	0.75 (0.58 - 0.96)
ER divergence	0.033	0.2	0.56	0.81 (0.66 - 0.98)
SNV divergence	0.086	0.43	0.58	1.4 (0.96 - 1.9)
CNA burden	0.12	0.48	0.54	1.2 (0.95 - 1.6)
ER intensity	0.12	0.48	0.51	1.2 (0.96 - 1.4)
GLUT1 intensity	0.22	0.48	0.54	1.1 (0.93 - 1.4)
GLUT1 divergence	0.23	0.48	0.54	0.88 (0.71 - 1.1)

Adj.p.val: adjusted p.value using the Holm correction. C: concordance. HR (95%CI): hazard ratio and 95% confidence interval for the standard score (z-scores) of the variable of interest.

Table S8. Univariate proportional hazard regressions of time to progression from nonprogressors

Variable	p.val	adj.p.val	C	HR (95% CI)
SNV burden	4.8e-05	0.00038	0.68	2.1 (1.5 - 3)
CNA burden	0.014	0.096	0.59	1.5 (1.1 - 2.1)
GLUT1 intensity	0.015	0.096	0.62	1.4 (1.1 - 1.9)
CNA divergence	0.026	0.13	0.59	0.67 (0.48 - 0.95)
ER intensity	0.076	0.3	0.54	1.3 (0.97 - 1.8)
ER divergence	0.57	1	0.54	0.93 (0.71 - 1.2)
SNV divergence	0.97	1	0.52	1 (0.59 - 1.7)
GLUT1 divergence	1	1	0.51	1 (0.75 - 1.3)

Adj.p.val: adjusted p.value using the Holm correction. C: concordance. HR (95%CI): hazard ratio and 95% confidence interval for the standard score (z-scores) of the variable of interest.

Table S9. Univariate proportional hazard regressions of time to non-invasive recurrence

Variable	p.val	adj.p.val	C	HR (95% CI)
SNV divergence	0.024	0.2	0.62	1.7 (1.1 - 2.8)
ER divergence	0.026	0.2	0.57	0.72 (0.54 - 0.96)
CNA divergence	0.038	0.23	0.6	0.68 (0.47 - 0.98)
GLUT1 divergence	0.17	0.87	0.55	0.79 (0.57 - 1.1)
ER intensity	0.19	0.87	0.52	1.2 (0.92 - 1.5)
SNV burden	0.21	0.87	0.55	1.2 (0.88 - 1.8)
CNA burden	0.4	0.87	0.54	1.2 (0.81 - 1.7)
GLUT1 intensity	0.94	0.94	0.5	0.99 (0.77 - 1.3)

Adj.p.val: adjusted p.value using the Holm correction. C: concordance. HR (95%CI): hazard ratio and 95% confidence interval for the standard score (z-scores) of the variable of interest.

Table S10. Univariate proportional hazard regressions of time to progression

Variable	p.val	adj.p.val	C	HR (95% CI)
SNV burden	7.1e-05	0.00057	0.67	2.2 (1.5 - 3.2)
CNA divergence	0.025	0.17	0.59	0.68 (0.48 - 0.95)
ER intensity	0.025	0.17	0.56	1.4 (1 - 1.9)
GLUT1 intensity	0.027	0.17	0.61	1.4 (1 - 1.9)
CNA burden	0.045	0.18	0.57	1.4 (1 - 2)
ER divergence	0.42	1	0.54	0.89 (0.67 - 1.2)
GLUT1 divergence	0.78	1	0.53	0.96 (0.7 - 1.3)
SNV divergence	0.97	1	0.48	0.99 (0.59 - 1.7)

Adj.p.val: adjusted p.value using the Holm correction. C: concordance. HR (95%CI): hazard ratio and 95% confidence interval for the standard score (z-scores) of the variable of interest.
